# Tpl2 is required for VEGF-A-stimulated signal transduction and endothelial cell function

**DOI:** 10.1242/bio.034215

**Published:** 2019-05-09

**Authors:** Gareth W. Fearnley, Izma Abdul-Zani, Antony M. Latham, Monica C. Hollstein, John E. Ladbury, Stephen B. Wheatcroft, Adam F. Odell, Sreenivasan Ponnambalam

**Affiliations:** 1 School of Molecular & Cellular Biology, University of Leeds, Leeds, LS2 9JT, UK; 2 Leeds Institute of Cardiovascular & Metabolic Medicine, University of Leeds, Leeds, LS2 9JT, UK; 3 Leeds Institute for Medical Research at St. James's, University of Leeds, Leeds, LS2 9JT, UK

**Keywords:** Endothelial, VEGF-A, Tpl2, Signal transduction, Angiogenesis

## Abstract

New blood vessel sprouting (angiogenesis) and vascular physiology are fundamental features of metazoan species but we do not fully understand how signal transduction pathways regulate diverse vascular responses. The vascular endothelial growth factor (VEGF) family bind membrane-bound receptor tyrosine kinases (VEGFRs), which trigger multiple signal transduction pathways and diverse cellular responses. We evaluated whether the MAP3K family member and proto-oncoprotein Tpl2 (MAP3K8) regulates basal and VEGF-A-stimulated signal transduction in endothelial cells. Notably, stimulation with exogenous VEGF-A increased *Tpl2* mRNA levels and consequently *de novo* protein synthesis. Depletion of Tpl2 levels reveals a role in both basal and VEGF-A-stimulated endothelial cell responses, including endothelial-leukocyte interactions, monolayer permeability and new blood vessel formation. Under basal conditions, Tpl2 modulates a signal transduction cascade resulting in phosphorylation of a nuclear transcription factor (ATF-2) and altered endothelial gene expression, a pathway previously identified as crucial in VEGF-dependent vascular responses. Loss of Tpl2 expression or activity impairs signal transduction through Akt, eNOS and ATF-2, broadly impacting on endothelial function. Our study now provides a mechanism for Tpl2 as a central component of signal transduction pathways in the endothelium.

## Abstract (precis)

**Summary:** Our study shows that the growth factor VEGF-A stimulates synthesis in endothelial cells of a proto-oncoprotein and protein kinase, Tpl2, and this is required for signal transduction and angiogenesis.

## INTRODUCTION

Growth factors are soluble substances that bind plasma membrane receptors and modulate cellular responses, tissue homeostasis, organ development and animal physiology. Receptor-ligand complexes exert such biological responses through activation of multiple signal transduction pathways, which modulate cell and tissue function. Receptor tyrosine kinases (RTKs) are a class of membrane-bound receptors that regulate many aspects of cell and tissue function in eukaryotes (Lemmon and Schlessinger, 2010). RTK function is frequently linked to the activation of a canonical mitogen-activated protein kinase (MAPK) pathway (Plotnikov et al., 2011). Different, parallel MAPK-like signal transduction pathways can utilize hierarchical arrays of closely related MAPK enzymes and opposing protein phosphatases to regulate discrete biological processes. Such cycles of phosphorylation and dephosphorylation regulate enzyme or protein activity within different intracellular locations impacting on metabolism, gene expression, cell migration and cell proliferation (Katz et al., 2007). Although many well-established signal transduction models exist from yeast to human, we still lack a full understanding of how MAPK signal transduction pathways regulate complex responses by primary cells or tissues.

The mammalian endothelium is a useful model for understanding how signal transduction regulates complex biological responses e.g. cell migration, cell proliferation and building vascular tubes (Lammert and Axnick, 2012; Simons and Eichmann, 2015). The endothelium is a terminally differentiated cell monolayer that lines all blood vessels and responds to extrinsic factors such as leukocytes, platelets, growth factors, lipids and pro-inflammatory mediators (Muller, 2009). This is exemplified by the family of vascular endothelial growth factors (VEGFs), which bind a specific subfamily of RTKs (VEGFR1, 2 and 3) expressed by the endothelium to regulate cell function, vascular and lymphatic development (Koch and Claesson-Welsh, 2012; Smith et al., 2015a). VEGF family members regulate biological processes such as vasculogenesis, angiogenesis and lymphangiogenesis. The founding member of this family, VEGF-A, binds VEGFR2 to activate multiple signal transduction pathways that regulate vasculogenesis and angiogenesis (Smith et al., 2015b). Such intracellular signaling is essential for normal vascular function but is subverted in tumor angiogenesis, a key feature of cancer initiation and progression (Jain, 2014; Kieran et al., 2012).

VEGF-A binding to VEGFR2 activates the canonical MAPK signal transduction pathway involving MEK1 and ERK1/2 (p42/44 MAPK) (Domigan et al., 2015; Simons and Eichmann, 2015; Smith et al., 2015b). Furthermore, VEGF-A also activates other signal transduction pathways leading to increased phosphorylation and activation of Akt and p38 MAPK enzymes, which in turn impact on nitric oxide levels, protein translation and cellular stress responses (Fearnley et al., 2014a, 2016). A well-established paradigm in cancer biology is that increased VEGF-A levels cause tumor neovascularization leading to tumor growth and metastasis (Jain, 2014; Kieran et al., 2012; Vasudev and Reynolds, 2014). However, blood vessels within tumors are often immature, tortuous, leaky and prone to collapse under the elevated interstitial fluid pressure. Normalization of the tumor vasculature represents a promising target for anti-cancer therapy development. However, this relies on identifying the critical signaling and transcriptional programs controlling VEGF-dependent vascular leakage. In this study, we investigated whether the Raf-related protein kinase, Tpl2 (MAP3K8), plays a role in MAPK signal transduction and endothelial function. We found that in primary human endothelial cells, VEGF-A stimulates Tpl2 synthesis. Further analysis suggests Tpl2 is functionally linked to MAPK signal transduction and nuclear gene expression, which in turn influence cellular responses relevant for angiogenesis and endothelial barrier integrity. Our study now suggests a central role for Tpl2 in regulating endothelial function.

## RESULTS

### VEGF-A regulates Tpl2 expression and endothelial cell responses

**Fig. 1.**
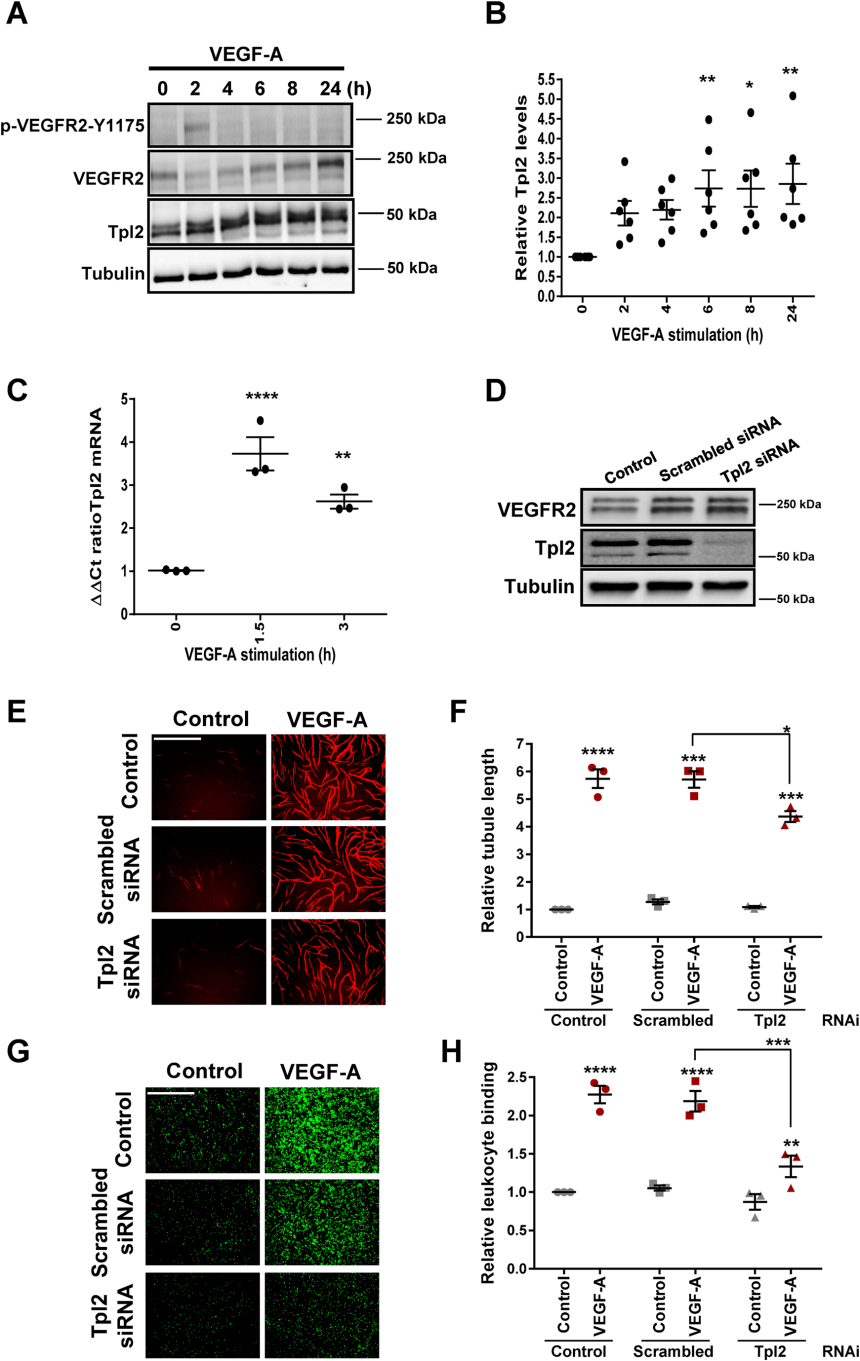
**VEGF-A-stimulated Tpl2 expression modulates endothelial cell responses.** (A) Endothelial cells stimulated with VEGF-A (25 ng/ml) for 2, 4, 6, 8 or 24 h were lysed and subjected to immunoblot analysis. (B) Quantification of VEGF-A-stimulated changes in relative Tpl2 protein levels using immunoblotting data. Error bars indicate ±s.e.m. (*n*=6). Statistical test=one-way ANOVA; *P<0.05, **P<0.01. (C) Endothelial cells stimulated with VEGF-A (25 ng/ml) for 1.5 or 3 h prior to quantification of relative *Tpl2* mRNA levels using qRT-PCR. Error bars indicate ±s.e.m. (*n*=3). Statistical test=one-way ANOVA; **P<0.01, ****P<0.0001. (D) Immunoblot analysis of control (mock-transfected), scrambled siRNA-treated or Tpl2-depleted endothelial cells (E–H). Control (mock-transfected), scrambled or Tpl2-depleted endothelial cells were stimulated with VEGF-A (25 ng/ml), processed with different assays (see Materials and Methods) and quantified for cellular responses in (E,F) endothelial tubulogenesis, or (G,H) endothelial-leukocyte interactions. Scale bars: 1000 µm. Error bars indicate ±s.e.m. (*n*=3). In panels F and H, statistical tests=two-way ANOVA; *P<0.05, **P<0.01, ***P<0.001, ****P<0.0001.

Gene expression profiling (https://www.ncbi.nlm.nih.gov/geoprofiles/) suggests that the VEGF-A may regulate Raf-related MAP3K family member and proto-oncogene, Tpl2 (MAP3K8) expression (Schoenfeld et al., 2004; Schweighofer et al., 2009) with more recent work implicating Tpl2 in angiogenesis (Lee et al., 2013). One likelihood, based on VEGF-A-stimulated gene profiling (Schoenfeld et al., 2004; Schweighofer et al., 2009) and *VCAM-1* expression (Fearnley et al., 2014a), is that VEGF-A-stimulated signal transduction promotes *Tpl2* gene transcription and protein expression. To test this idea, we stimulated primary human umbilical vein endothelial cells (HUVECs) with VEGF-A for different time periods and monitored Tpl2 protein levels in relation to controls (Fig. 1A). Here, VEGF-A stimulation caused a relatively rapid ∼2.5-fold increase in Tpl2 protein levels (doublet of 52 and 58 kDa) within 2 h and this effect was sustained over a 24 h period (Fig. 1B). Analysis of mRNA levels revealed that VEGF-A stimulation caused either ∼3.5-fold (1.5 h) or ∼2.5-fold (3 h) increase in *Tpl2* mRNA levels (Fig. 1C), preceding the changes in Tpl2 protein levels.

VEGF-A stimulation of endothelial cells promotes distinct patterns of gene expression and cellular responses (Fearnley et al., 2014a; Matsumoto and Claesson-Welsh, 2001; Neagoe et al., 2005). To assess whether Tpl2 plays a role in such VEGF-A-regulated responses, we used RNA interference (RNAi) to assess the functional requirement for Tpl2. Small inhibitory RNA (siRNA) duplex-mediated knockdown caused noticeable decrease in Tpl2 protein levels (Fig. 1D) corresponding to ∼60% reduction (Fig. S1A). Knockdown of Tpl2 caused an inhibition in VEGF-A-stimulated responses including ∼35% decrease in endothelial tubulogenesis (Fig. 1E,F) and ∼45% decrease in endothelial–leukocyte interactions (Fig. 1G,H). Furthermore, Tpl2 knockdown caused ∼20% decrease in endothelial cell viability (Fig. S1B). These data suggest that Tpl2 regulates both cell viability and VEGF-A-stimulated endothelial responses.

### Tpl2 levels regulate ATF-2 phosphorylation, turnover and function

**Fig. 2.**
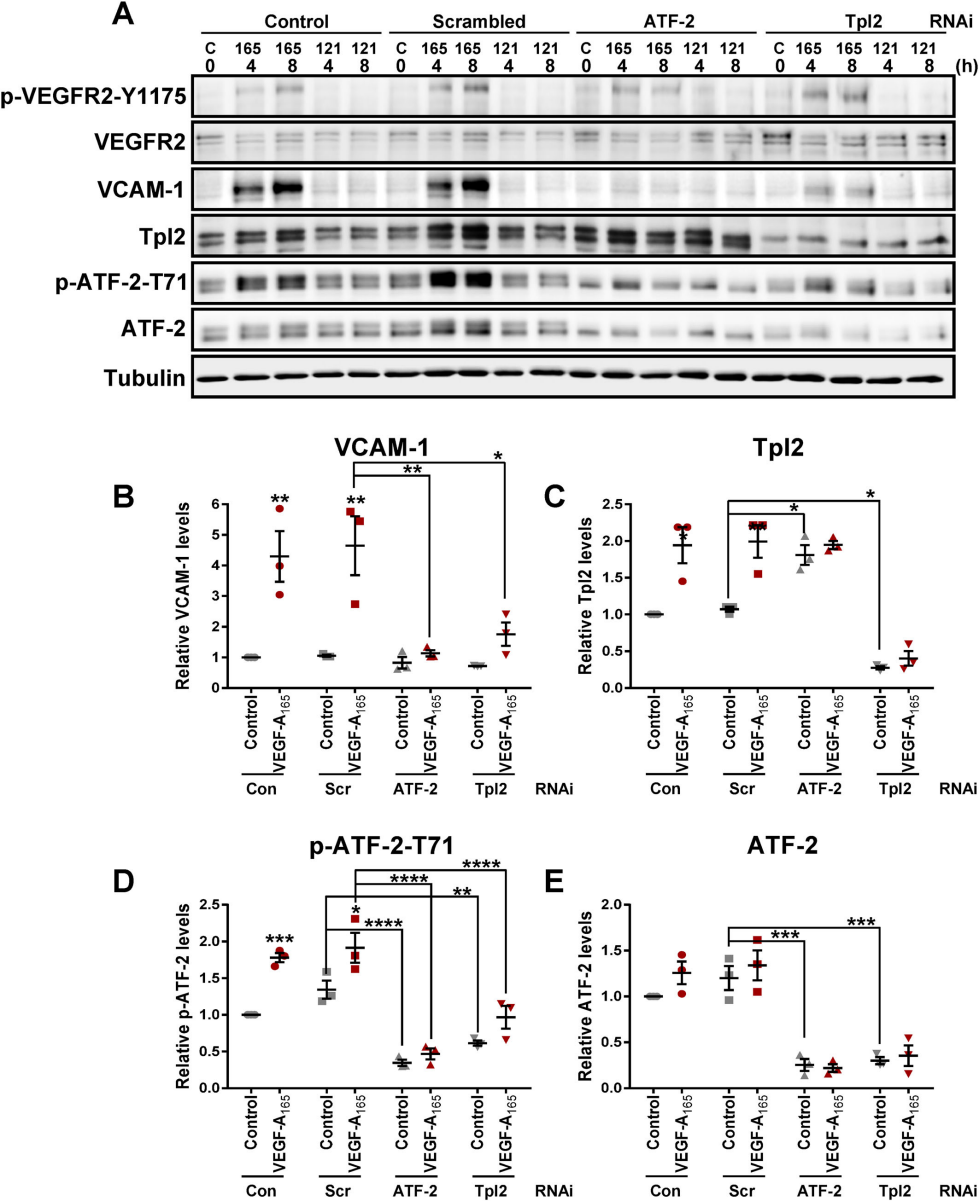
**Tpl2 is required for VEGF-A-stimulated ATF-2-dependent VCAM-1 expression.** (A) Control, scrambled, Tpl2 or ATF-2-specific siRNA-treated endothelial cells were stimulated (25 ng/ml) with either VEGF-A_165_ or VEGF-A_121_ (negative control) for 4 or 8 h, prior to immunoblot analysis. (B–E) Quantification of protein levels by immunoblot analyses (see the Materials and Methods) of VEGF-A (25 ng/ml) stimulated control, scrambled, Tpl2-depleted or ATF-2 depleted endothelial cells for protein levels of (B) VCAM-1, (C) Tpl2, (D) ATF-2-pT71 and (E) ATF-2. Error bars indicate ±s.e.m. (*n*=3). In panels B–E, statistical tests=two-way ANOVA; *P<0.05, **P<0.01, ***P<0.001, ****P<0.0001.

Tightly regulated expression of vascular cell adhesion molecule 1 (VCAM-1) on endothelial cells influences leukocyte binding to the activated endothelium (Nourshargh et al., 2010). Previous studies have shown that VEGF-A stimulates endothelial VCAM-1 expression via the canonical MEK1-ERK1/2 signaling pathway and hyperphosphorylation of nuclear activating transcription factor 2 (ATF-2) (Fearnley et al., 2014a). Knockdown of Tpl2 levels and the significant decrease in VEGF-A-stimulated endothelial-leukocyte interactions (Fig. 1G,H) raised the possibility that Tpl2 regulates the signal transduction pathway leading to ATF-2 hyperphosphorylation and VCAM-1 expression. To address this, RNAi was used to knockdown either endothelial ATF-2 or Tpl2 prior to stimulation with two VEGF-A isoforms (VEGF-A_165_ and the less bioactive VEGF-A_121_) followed by immunoblotting (Fig. 2). VEGF-A_165_ but not VEGF-A_121_ stimulation increased VCAM-1 protein levels after 4 h (Fig. 2A). Consequently, we focused on VEGF-A_165_-stimulated endothelial responses for further investigation.

Elevated VCAM-1 levels were associated with the presence of phosphorylated ATF-2 (p-ATF-2) and Tpl2 as depletion of either ATF-2 or Tpl2 prevented VEGF-A-stimulated increases in VCAM-1 expression (Fig. 2A). Although VEGF-A stimulation caused a >four-fold rise in VCAM-1 levels (Fig. 2B), Tpl2 knockdown reduced VCAM-1 expression close to baseline levels (Fig. 2B). This effect was comparable to the decreased VCAM-1 levels caused by ATF-2 depletion (Fig. 2B). Notably, ATF-2 depletion also caused ∼two-fold increase in Tpl2 levels under basal conditions in the presence of relatively low VEGF-A levels (≤0.5 ng/ml) with loss of VEGF-A-stimulated increase in Tpl2 levels (Fig. 2C). Surprisingly, analysis of phospho-ATF-2 (pT71) showed that Tpl2 depletion caused a significant decrease in basal and VEGF-A-stimulated levels (Fig. 2D). There was a similar ∼70% in total ATF-2 levels (Fig. 2E). Such findings suggested a functional link between Tpl2 and ATF-2.

**Fig. 3.**
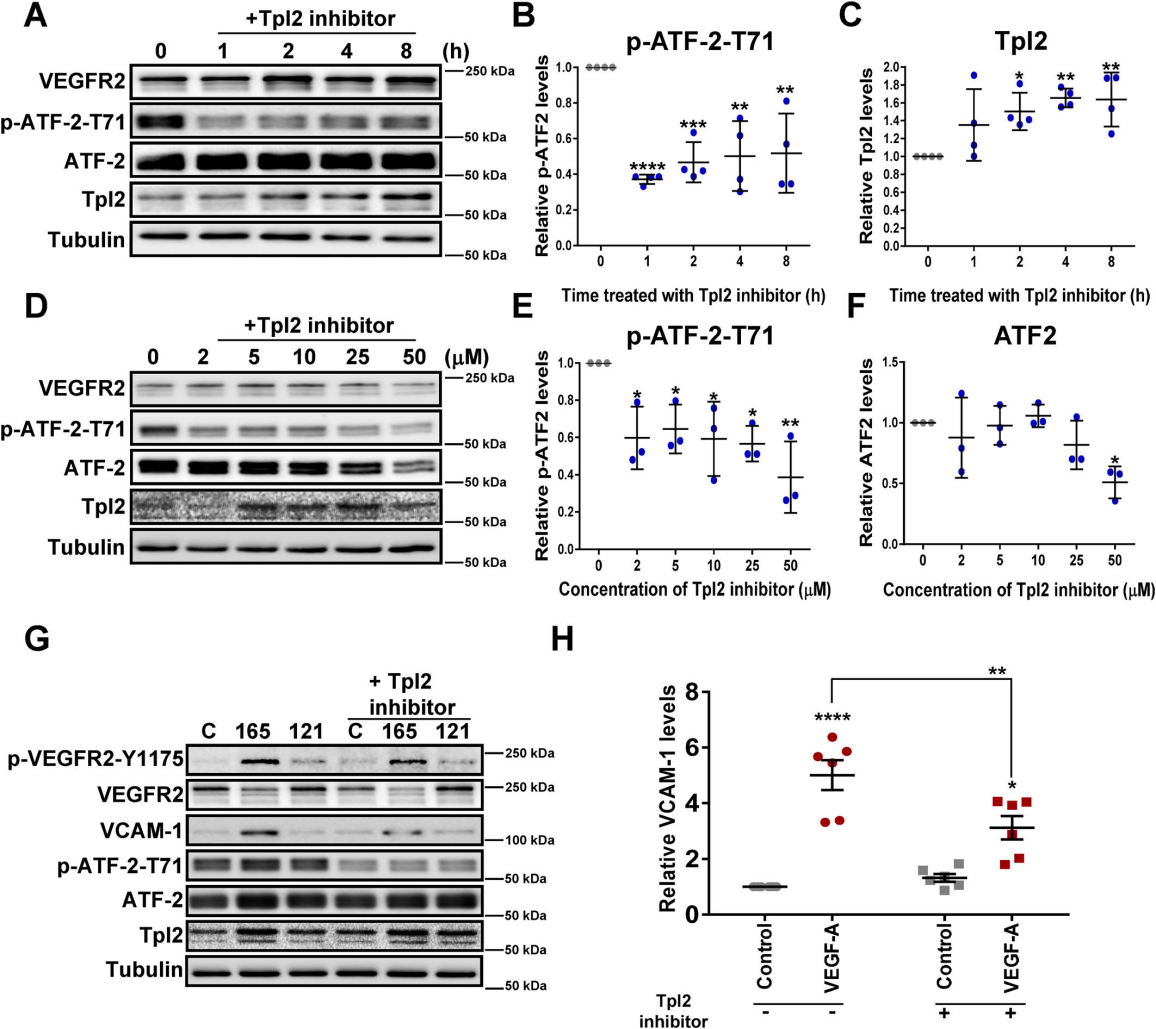
**Tpl2-selective kinase inhibitor treatment attenuates ATF-2 phosphorylation and expression levels.** (A–F) Analysis of basal endothelial signaling in non-stimulated cells. (A) Immunoblot analysis of endothelial cells treated with Tpl2-selective kinase inhibitor (2 µM) for 0, 1, 2, 4 or 8 h to assess effects on VEGFR2, ATF-2 and Tpl2 status. Quantification of immunoblot data for Tpl2-selective inhibitor time course for (B) ATF-2-pT71, and (C) Tpl2 levels. Error bars indicate ±s.e.m. (*n*=4). In panels B and C, statistical tests=one-way ANOVA; *P<0.05, **P<0.01, ***P<0.001, ****P<0.0001. (D) Immunoblot analysis of endothelial cell response to Tpl2-selective kinase inhibitor titration (2–50 µM, 4 h). (E,F) Quantification of immunoblot data from Tpl2-selective kinase inhibitor titration on (E) ATF-2-pT71, and (F) ATF-2 levels. Error bars indicate ±s.e.m. (*n*=3). In panels E and F, statistical tests=one-way ANOVA; *P<0.05, **P<0.01. (G) Endothelial cells were pre-treated with a Tpl2-selective kinase inhibitor (2 µM; 30 min) prior to stimulation with either (25 ng/ml) VEGF-A_165_ (165) or VEGF-A_121_ (121, negative control) for 0 or 8 h before lysis and immunoblot analysis. (H) Quantification of immunoblot data showing changes in relative VCAM-1 levels upon VEGF-A (25 ng/ml) treatment in the absence (−) or presence (+) of Tpl2 kinase inhibitor. Error bars indicate ±s.e.m. (*n*=6). Statistical test=two-way ANOVA; *P<0.05, **P<0.01, ****P<0.0001.

To further investigate how Tpl2 and ATF-2 were linked, we treated endothelial cells with the small molecule Tpl2-selective kinase inhibitor CAS 871307-18-5 (IC_50_ ∼50 nM) and assessed effects on signal transduction and VCAM-1 expression (Fig. 3). Endothelial cells were subjected to a time course of treatment with 2 µM Tpl2-selective kinase inhibitor (Fig. 3A), prior to quantification of relative levels of phospho-ATF-2 (Fig. 3B) and Tpl2 (Fig. 3C). Treatment with Tpl2-selective kinase inhibitor caused a relatively rapid reduction in ATF-2-pT71 levels but did not affect total ATF-2 levels (Fig. 3A); this ∼60% decrease in phospho-ATF2 persisted up to 8 h (Fig. 3B). Tpl2-selective kinase inhibitor treatment also caused a gradual rise in Tpl2 levels (Fig. 3A,C) with ∼60% increase in Tpl2 levels evident after 4 h (Fig. 3C). We then tested increasing concentrations of Tpl2-selective kinase inhibitor for 4 h and again found that ATF-2-pT71 levels were substantially reduced (Fig. 3D,E). Notably, total ATF-2 levels were substantially depleted by ∼50% when Tpl2-selective kinase inhibitor concentration was >25 µM (Fig. 3D,F).

VEGF-A-stimulated phosphorylation of ATF-2 correlates with elevated VCAM-1 expression (Fig. 2D versus B). Furthermore, Tpl2 depletion impaired VEGF-A-stimulated VCAM-1 expression (Fig. 2B). To investigate the link between Tpl2 kinase activity and VEGF-A-stimulated signaling, we pre-treated endothelial cells with Tpl2-selective kinase inhibitor, (2 µM, 30 min) prior to VEGF-A isoform stimulation (Fig. 3G). Here, treatment with Tpl2-selective kinase inhibitor caused a clear reduction in VEGF-A-stimulated VCAM-1 expression (Fig. 3G) corresponding to a ∼38% decrease in VCAM-1 levels (Fig. 3H). These data show Tpl2 requirement in both ATF-2 phosphorylation and VEGF-A-stimulated VCAM-1 expression.

### Tpl2 is functionally linked to angiogenesis

**Fig. 4.**
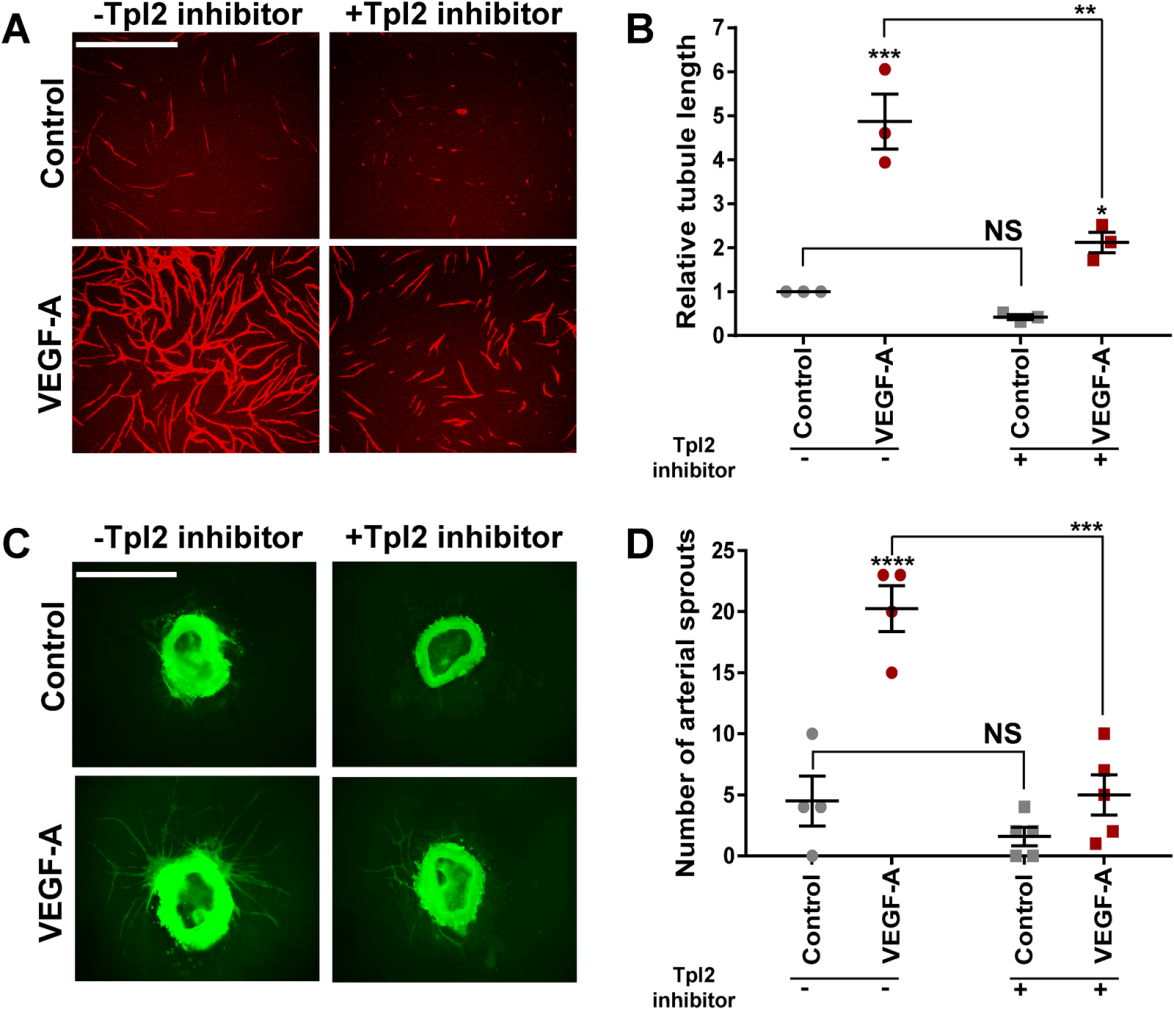
**Tpl2-selective kinase inhibitor treatment impairs VEGF-A-stimulated endothelial cell tubulogenesis and aortic sprouting.** (A) Endothelial cells co-cultured with primary human fibroblasts were stimulated with VEGF-A (25 ng/ml)±Tpl2-selective kinase inhibitor (2 µM). Endothelial cell tubules were fixed and stained for the Tpl2-selective kinase endothelial cell-specific marker and cell adhesion molecule (PECAM-1) for visualization using fluorescence microscopy. (B) Quantification of endothelial tubule length from fluorescence microscopy datasets. Error bars indicate ±s.e.m. (*n*=3). Statistical test=two-way ANOVA; *P<0.05, **P<0.01, ***P<0.001. (C) Endothelial sprouting from mouse aortic slices stimulated with VEGF-A (25 ng/ml)±2 µM Tpl2-selective kinase inhibitor followed by fixation, staining with FITC-conjugated endothelial-specific lectin (see the Materials and Methods) and visualization using fluorescence microscopy. (D) Quantification of endothelial sprout number from mouse aortas upon treatment with VEGF-A±Tpl2, selective kinase inhibitor. Error bars indicate ±s.e.m. (*n*≥4). Statistical test=two-way ANOVA; ***P<0.001, ****P<0.0001. Scale bars: 1000 µm.

Our initial findings presented in this study (Fig. 1) indicated Tpl2 expression is required for VEGF-A-regulated endothelial tubulogenesis, a key feature of angiogenesis. To determine the effects of the Tpl2-selective kinase inhibitor on VEGF-A-stimulated endothelial tubulogenesis and aortic sprouting (Fig. 4). Here, Tpl2-selective kinase inhibitor treatment caused a substantial reduction in VEGF-A-stimulated endothelial tubulogenesis (Fig. 4A) corresponding to ∼50% decrease in VEGF-A-stimulated endothelial tubule length (Fig. 4B). Using a mouse aortic sprouting assay, Tpl2-selective kinase inhibitor also reduced VEGF-A-stimulated endothelial sprout formation (Fig. 4C) with a ∼five-fold reduction (Fig. 4D). Treatment with Tpl2-selective kinase inhibitor had no significant effect on non-stimulated (basal) tubulogenesis (Fig. 4B) or aortic sprouting in the absence of VEGF-A (Fig. 4D). These data further support a role for Tpl2 in regulating VEGF-A-stimulated angiogenesis.

### Tpl2 regulates signal transduction and endothelial cell permeability

Endothelial cell permeability is a tightly controlled process for normal vascular physiology and can combat pathological conditions e.g. blood vessel damage (Mehta and Malik, 2006; Trani and Dejana, 2015). One regulatory mechanism that controls vascular permeability is VEGF-A-regulated activation of endothelial nitric oxide synthase (eNOS) (Lal et al., 2001; Six et al., 2002). VEGF-A-stimulated signal transduction promotes Akt phosphorylation and elevated Akt kinase activity, which in turn phosphorylates and activates eNOS leading to elevated NO levels (Dimmeler et al., 1999; Lal et al., 2001; Six et al., 2002). The NO molecule acts as a rapidly diffusible second messenger that promotes endothelial monolayer permeability (Di Lorenzo et al., 2013; Lal et al., 2001; Six et al., 2002).

**Fig. 5.**
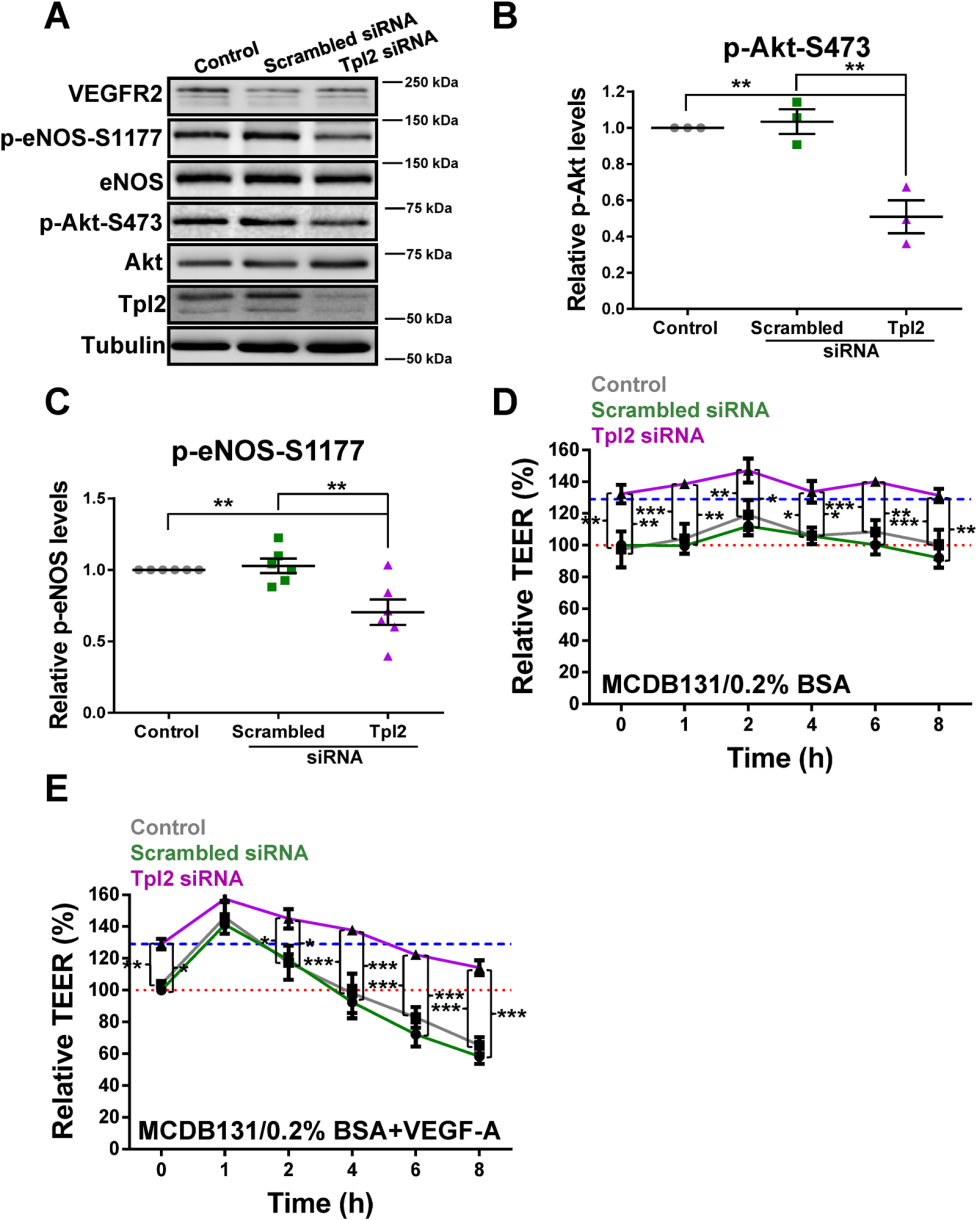
**Tpl2 modulates eNOS phosphorylation and endothelial cell permeability.** (A) Immunoblot analysis of control, scrambled or Tpl2-specific siRNA-treated endothelial cells to assess VEGFR2, eNOS, Akt and Tpl2 status. Quantification of immunoblot data for levels of (B) Akt-pS473 and (C) eNOS-pS1177 levels. Error bars indicate ±s.e.m. (*n*≥3). In panels B and C, statistical tests=one-way ANOVA; **P<0.01. (D–E) Assessment of endothelial cell permeability by measuring trans-endothelial electrical resistance (TEER) on control, scrambled or Tpl2-depleted endothelial cells under (D) basal conditions in serum-free MCDB131 media, or (E) with addition of VEGF-A (25 ng/ml). High TEER values correspond to reduced cell monolayer permeability indicative of more stable cell–cell junctions. Horizontal red dotted and blue dashed lines depict baselines for control/scrambled or Tpl2-depleted cells respectively. Error bars indicate ±s.e.m. (*n*=3). In panels D and E, statistical tests=two-way ANOVA; *P<0.05, **P<0.01, ***P<0.001.

Tpl2 kinase activity is implicated in Akt phosphorylation and activation (López-Peláez et al., 2011; Li et al., 2015), suggesting that endothelial Tpl2 could regulate eNOS status and endothelial monolayer permeability. To investigate this further, we analyzed Akt and eNOS phosphorylation and overall levels in control or Tpl2-depleted endothelial cells (Fig. 5A). Quantification of these data showed that Tpl2 depletion caused a ∼50% decrease in relative levels of phospho-Akt (Akt-pS473) (Fig. 5B). Furthermore, Tpl2 depletion also caused a ∼30% decrease in relative levels of phospho-eNOS (Fig. 5C). To check for the influence of other signal transduction pathways, small molecule kinase inhibitors specific for ERK1/2 (PD98059; IC_50_, 1-20 µM), p38 MAPK (SB203580; IC_50_, 500 nM), JNK (SP600125; IC_50_, 100-150 nM) or Akt (MK2206; IC_50_, 8-65 nM) were tested and phospho-Akt and phospho-eNOS levels evaluated (Fig. S2A). Quantification of these data showed that Akt inhibition by MK2206 caused a ∼40% decrease in relative levels of phospho-eNOS compared to control; pharmacological inhibition of EKR1/2, p38 MAPK or JNK had little or no effect (Fig. S2B). Thus under basal conditions, Tpl2 influences Akt and eNOS phosphorylation.

To assess whether Tpl2-mediated regulation of Akt and eNOS status had a functional effect on endothelial cell permeability, we measured a parameter called trans-endothelial electrical resistance (TEER). Under basal (non-stimulated) conditions, Tpl2-depleted endothelial cells exhibited a ∼30% increase in relative TEER value (indicating decreased cell monolayer permeability) compared to control (Fig. 5D). Upon VEGF-A stimulation however, we observed a time-dependent oscillation in relative TEER values (Fig. 5E). Within 1 h of VEGF-A stimulation, endothelial cells exhibited a transient rise in relative TEER values for control, scrambled and Tpl2 siRNA-treated endothelial cells (Fig. 5E). This was followed by a gradual decrease in TEER values over the next 7 h of VEGF-A treatment, consistent with an increase in endothelial cell permeability (Fig. 5E). The decrease in TEER values was more pronounced in controls and scrambled siRNA-treated cells over time, compared to Tpl2-depleted endothelial cells (Fig. 5E). Thus Tpl2 contributes to VEGF-A-stimulated increase in endothelial monolayer permeability.

**Fig. 6.**
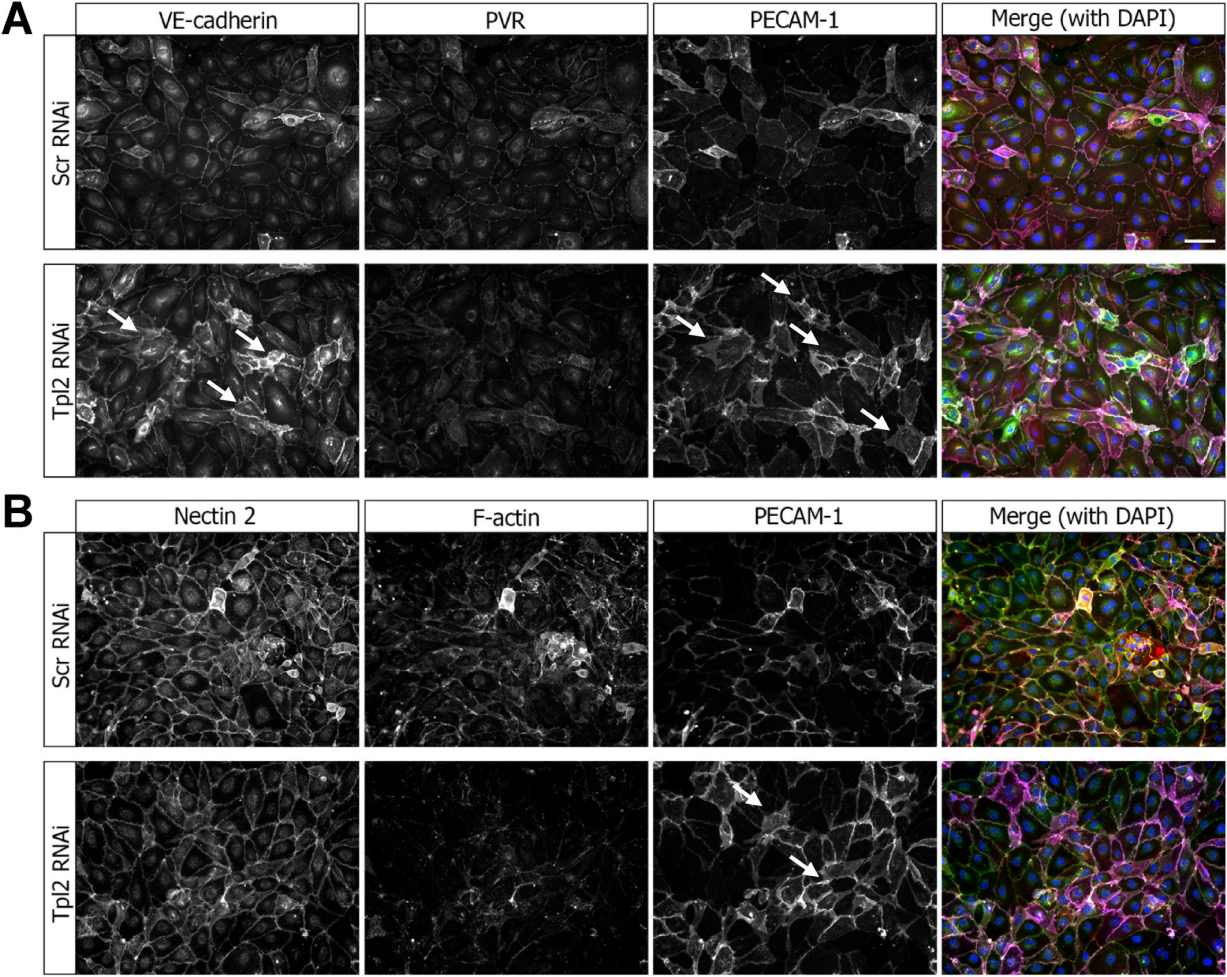
**Tpl2 modulates endothelial cell adhesion.** Endothelial cells were subjected to controls scrambled siRNA duplex treatment (Scr RNAi) or Tpl2-specific siRNA duplex (Tpl2 RNAi) and analyzed by confocal laser microscopy. (A) Endothelial cells subjected to RNAi were fixed and stained for cell-surface adhesion proteins such as VE-cadherin, PVR and PECAM-1. Arrows indicate cells showing increased staining for VE-cadherin and PECAM-1 under conditions of Tpl2 knockdown. Scale bar: 20 µm. (B) Endothelial cells subjected to RNAi were fixed and stained for Nectin 2, PVR and PECAM-1. Arrows indicate cells showing increased staining for F-actin and PECAM-1 upon Tpl2 knockdown.

Importantly, Tpl2 depletion caused increased expression of endothelial cell junctional proteins VE-cadherin and PECAM-1 (Fig. 6A). However, there were no significant changes in expression of another endothelial cell surface marker and adhesion protein, PVR (CD155), upon Tpl2 depletion (Fig. 6A). Nectin 2 is another cell surface membrane glycoprotein that is part of the adherens junction complex: Tpl2 depletion did not significantly affect expression of this protein (Fig. 6B). Under conditions of Tpl2 depletion, and elevated PECAM-1 levels, there was some changes in F-actin staining (Fig. 6B).

**Fig. 7.**
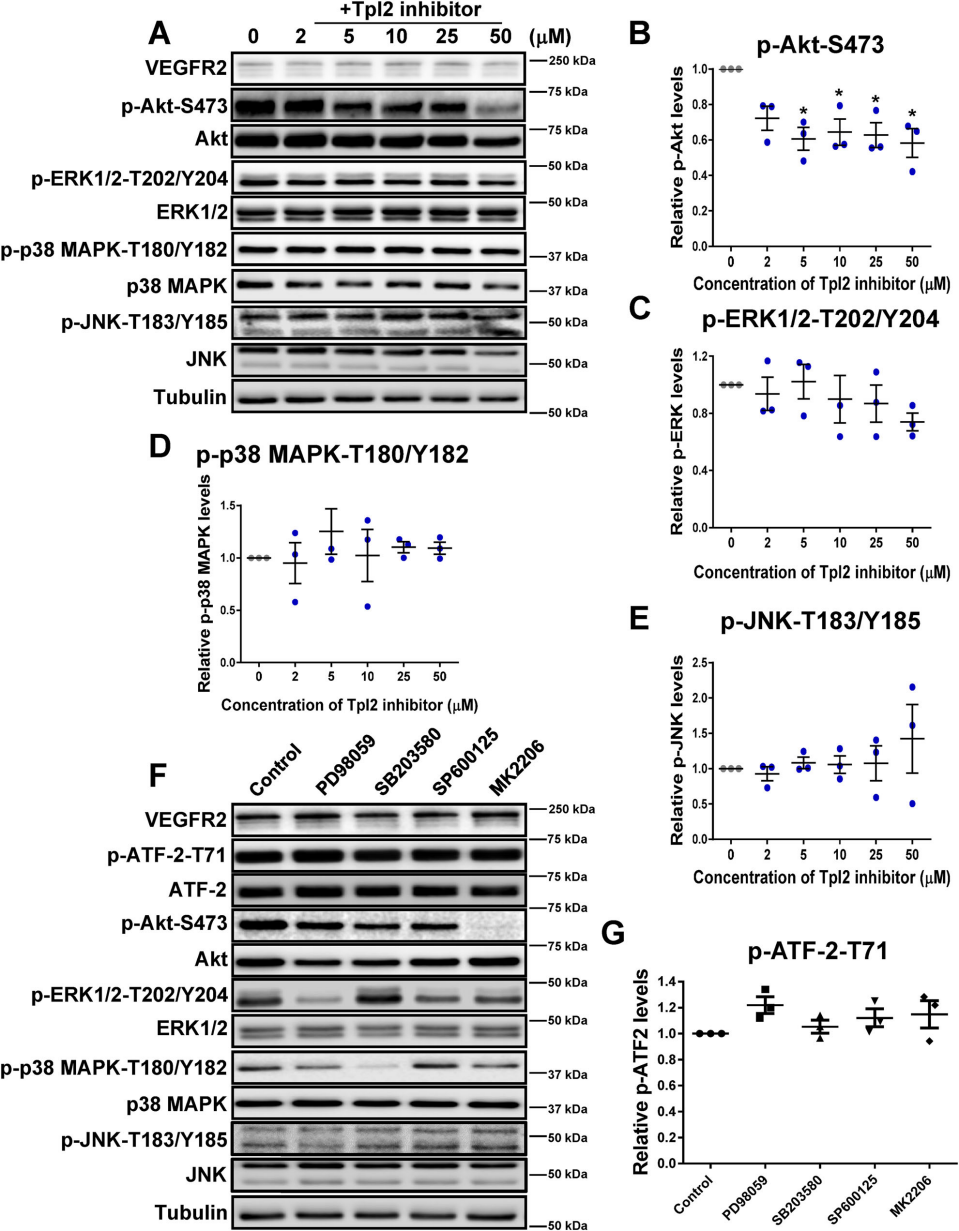
**Probing the link between Tpl2, Akt activation and ATF-2 status.** (A) Immunoblot analysis of endothelial cells titrated with Tpl2-selective kinase inhibitor (2–50 µM, 4 h). Quantification of immunoblot data upon inhibitor titration on activated kinases such as (B) Akt-pS473, (C) ERK1/2-pT202/pY204, (D) p38 MAPK-pT180/pY182 and (E) JNK-pT183/pY185. Error bars indicate ±s.e.m. (*n*=3). In panels B–E, statistical tests=one-way ANOVA; *P<0.05. (F) Immunoblot analysis of endothelial cells treated with small molecule kinase inhibitors (2 µM, 30 min) specific for ERK1/2 (PD98059), p38 MAPK (SB203580), JNK (SP600125) and Akt (MK2206). (G) Quantification of immunoblot data for relative ATF-2-pT71 levels upon inhibition of different protein kinases. Error bars indicate ±s.e.m. (*n*=3).

It is feasible that Tpl2 regulates multiple signal transduction pathways. This could be relevant in this study as multiple signal transduction pathways including ERK1/2, p38 MAPK and JNK are implicated in ATF-2 phosphorylation (Lau and Ronai, 2012). To assess whether other signaling pathways are linked to Tpl2 regulated ATF-2 hyperphosphorylation and turnover, we assessed whether Tpl2-selective kinase inhibitor titration affected ERK1/2, p38 MAPK, JNK and Akt phosphorylation (Fig. 7A). As previously observed, Tpl2-selective kinase inhibitor caused a 30–40% reduction in phospho-Akt levels (Fig. 7B) but no significant effects on ERK1/2, p38 MAPK or JNK phosphorylation were evident (Fig. 7C–E). We then assessed whether small molecule inhibitors specific for ERK1/2 (PD98059), p38 MAPK (SB203580), JNK (SP600125) or Akt (MK2206) affected ATF-2 status (Fig. 7F). These inhibitors blocked target kinase phosphorylation as expected but did not affect ATF-2-pT71 or ATF-2 levels (Fig. 7F). Quantification of these data confirmed inhibition of these different signal transduction pathways did not significantly affect phospho-ATF-2 levels (Fig. 7G).

## DISCUSSION

**Fig. 8.**
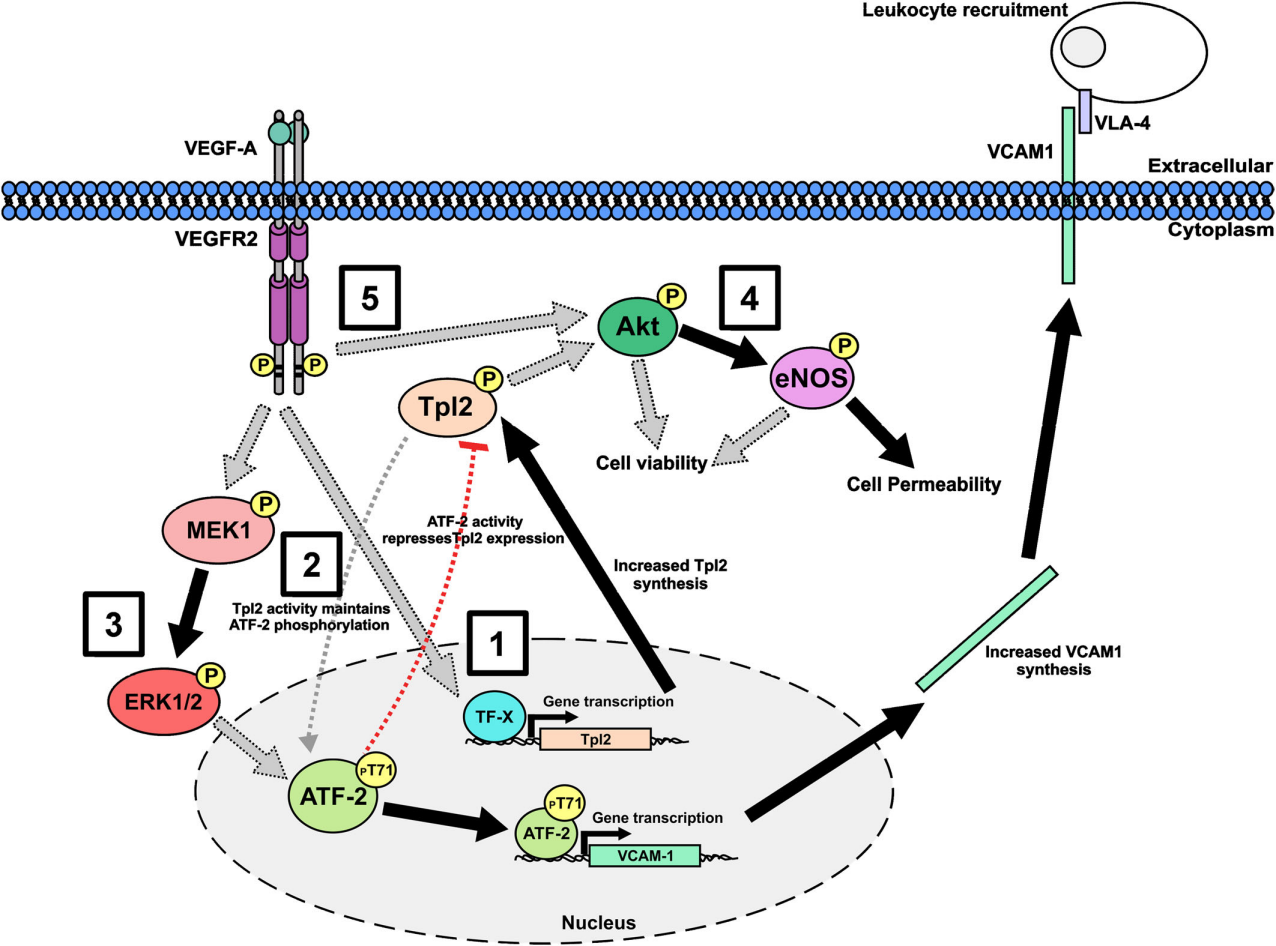
**Schematic showing a role for Tpl2 in endothelial function the cellular response to VEGF-A.** Step 1: VEGF-A stimulation promotes increased Tpl2 expression and activation via an unknown transcription factor (TF-X). Step 2: Tpl2 maintains phosphorylation of residue T71 on ATF-2 stabilizes ATF-2 levels. Step 3: VEGF-A-stimulated hyperphosphorylation of residue T71 on ATF-2 stimulates *VCAM-1* gene transcription leading to increased VCAM-1 levels and increased endothelial-leukocyte interaction. Step 4: basal Tpl2 activity promotes phosphorylation of residue S473 on Akt, causing Akt activation leading to eNOS-S1177 phosphorylation. Step 5: eNOS phosphorylation and activation leads to NO production and increased endothelial cell permeability. Grey arrows depict undefined mechanism.

The endothelial response to soluble factors such as VEGF-A regulates vasculogenesis and angiogenesis, important processes in health and disease (Jain, 2014; Smith et al., 2015b). Our study investigated the requirement for the proto-oncoprotein and MAP3K family member, Tpl2 (MAP3K8), in signal transduction and associated cellular responses in primary endothelial cells. Our findings now provide a role for Tpl2 in integrating multiple signal transduction pathways in regulating endothelial function (Fig. 8).

### Tpl2 is required for VEGF-A-stimulated endothelial cell responses

A central finding of our study is that VEGF-A-stimulated signal transduction pathways are linked to Tpl2 in endothelial cell responses. First, Tpl2 depletion reduces VEGF-A-stimulated endothelial tubulogenesis, which is a key requirement for angiogenesis. Second, treatment with a Tpl2-selective kinase inhibitor impaired both VEGF-A-stimulated endothelial tubulogenesis and aortic sprouting, further supporting functional requirement for Tpl2 in VEGF-A-stimulated angiogenesis. Third, Tpl2 depletion reduced endothelial-leukocyte interactions, showing Tpl2 contributes to the regulation of cell–cell interactions (Fig. 8). Finally, Tpl2 levels modulated Akt and eNOS activity, which affected monolayer permeability (Fig. 8). Our findings implicate Tpl2 as a key signaling component in endothelial cells, which impact on VEGF-A-stimulated angiogenesis and vascular control. This study complements previous observations that pharmacological inhibition of Tpl2 impairs tumor-associated angiogenesis*,* blood vessel formation *in vivo* and VEGF-A-stimulated endothelial cell responses (Lee et al., 2013).

### Tpl2 requirement in in basal signal transduction pathways

Our study provides several lines of evidence for Tpl2 regulation of basal signal transduction events impacting on endothelial cell function and homeostasis. Firstly, Tpl2 depletion or treatment with a Tpl2-selective inhibitor reduces both phospho- and steady-state ATF-2 levels, with a concomitant loss in VEGF-A-stimulated VCAM-1 expression. Previously it had been shown that VEGF-A and ATF-2 were part of a signaling pathway that controls VCAM-1 expression (Fearnley et al., 2014a), thus arguing that VEGF-A, Tpl2, ATF-2 and VCAM-1 are functionally linked to control signal transduction and gene expression. How Tpl2 modulates ATF-2 phosphorylation is unclear; however, one explanation is that Tpl2 phosphorylates and activates ERK1/2 (Banerjee et al., 2006; Dumitru et al., 2000), and phospho-ERK1/2 targets T71 residue on ATF-2 for phosphorylation (Lau and Ronai, 2012). Phosphorylation of T71 on ATF-2 blocks ubiquitination and subsequent degradation by the 26S proteasome (Fuchs et al., 2000), thus stabilizing its steady state levels. However, pharmacological inhibition of ERK1/2 has little effect on ATF-2-pT71 levels, suggesting differences in endothelial Tpl2 regulation of such events. Alternatively, as Tpl2 can be detected in the nucleus (Kanellis et al., 2015), activated Tpl2 could directly phosphorylate nuclear ATF-2. Our model provides a role for the Tpl2 kinase in communicating to both nuclear (ATF-2) and cytoplasmic effectors (e.g. Akt) (Fig. 8).

Many studies have documented the close link between Tpl2 and NF-κB with conflicting views as to whether Tpl2 activates or suppresses NF-κB activation (Vougioukalaki et al., 2011). Interleukin 1β activates endothelial NF-κB whereas VEGF-A does not behave similarly (Schweighofer et al., 2007); however, other studies suggest that VEGF-A does indeed activate NF-κB (Maeng et al., 2006). Nonetheless, genetic ablation of Tpl2 in astrocytes down-regulates VCAM-1 expression in response to ligands such as TNFα and bacterial lipopolysaccharide that classically activate the NF-κB pathway (Sriskantharajah et al., 2014), suggesting that Tpl2 and ATF-2 may be functionally linked in other cell types.

Our study shows that Tpl2 levels modulate both Akt and eNOS phosphorylation and activation, which further impacts on resting and VEGF-A-stimulated endothelial monolayer permeability. Depletion of Tpl2 levels causes a rise in relative TEER values, indicative of decreased endothelial permeability. Our finding that Tpl2 depletion causes elevated expression of adhesion proteins such as VE-cadherin and PECAM-1, key regulators of endothelial cell–cell monolayer integrity, could explain the increased cell–cell adhesion and decreased permeability. Interestingly, macrophages from *Tpl2*-null mice display reduced phospho-Akt levels (López-Peláez et al., 2011) and similarly in the liver (Li et al., 2015). Our studies provide the basis for functionally linking Tpl2 to surface adhesion proteins that regulate endothelial monolayer integrity and permeability.

### VEGF-A-regulated Tpl2 synthesis promotes signal transduction and cellular responses

One finding from our study is that exogenous VEGF-A stimulates new Tpl2 synthesis with a four-fold rise in *Tpl2* mRNA levels promoting a 2.5-fold rise in Tpl2 protein levels. Our work now shows that resting or basal Tpl2 levels influences phosphorylation of Akt and ATF-2 and downstream targets such as eNOS and the nuclear *VCAM1* locus respectively. As elevated levels of exogenous VEGF-A stimulation can promote new Tpl2 synthesis this could then potentiate the endothelial response to diverse ligands such as VEGF-A, CXCL1 or bFGF (Lee et al., 2013). Different VEGF-A isoforms can bind the VEGFR2 receptor tyrosine kinase and activate multiple signal transduction pathways with different cellular responses (Fearnley et al., 2016). Previous work showing that VEGF-A isoform-specific signal transduction via the canonical MEK1-ERK1/2 pathway causes hyperphosphorylation of ATF-2 linked to VCAM-1 expression (Fearnley et al., 2014a). It is well-established that the *VEGFA* gene encodes multiple isoforms which have significant differences in signaling properties and cellular responses (Fearnley et al., 2015, 2014a, 2016; Harper and Bates, 2008; Kawamura et al., 2008a, 2008b; Pan et al., 2007).

### VEGF-A and Tpl2 in disease

There is much debate on whether Tpl2 functions as a proto-oncoprotein and/or tumor suppressor (Lee et al., 2015; Vougioukalaki et al., 2011). Our study shows that Tpl2 has basal and ligand-stimulated properties which could potentially have different consequences in health or disease. A recent study suggests that Tpl2 levels are strongly linked to colorectal cancer (CRC) (Pyo et al., 2018). CRC is a disease strongly linked to dysregulation of the VEGF-VEGFR axis, with therapies aimed at targeting both soluble VEGF-A (Los et al., 2007) and membrane-bound VEGFRs (Morabito et al., 2006). A high proportion (∼40%) of CRC patients displayed high Tpl2 levels and frequent distant metastases (Pyo et al., 2018). These lines of evidence suggest that a functional link between VEGF-A and Tpl2 regulates serious disease states such as CRC.

Tpl2 kinase inhibitors can have therapeutic capability in pro-inflammatory disorders such as rheumatoid arthritis and inflammatory bowel disease (Gantke et al., 2011; Hall et al., 2007; Vougioukalaki et al., 2011). As our study suggests that endothelial Tpl2 is required for multiple aspects of endothelial cell function, homeostasis and angiogenesis, therapeutic strategies based on pharmacological inhibition of Tpl2 need caution. Despite using a combination of siRNA and pharmacological inhibition to validate our results, we do see some discrepancies between the two different approaches. In this context, single siRNA duplex use or rescue experiments using an RNAi-resistant Tpl2 construct could provide more insights. Furthermore, the current study lacks a Tpl2 kinase assay to confirm that the Tpl2-selective inhibitor was indeed targeting Tpl2 kinase activity within the cell. Further work is thus required to strengthen the importance of Tpl2 as a central regulator of endothelial responses. Nonetheless, our findings now provide a model for endothelial Tpl2 function (Fig. 8) which could be useful for better understanding cancer and immune dysfunction.

## MATERIALS AND METHODS

### Antibodies, reagents and growth factors

Antibodies: goat-anti-VEGFR2 cat. #AF357, mouse-anti-VCAM-1 cat. #BBA5 (R&D Systems, Minneapolis, MN, USA), goat-anti-nectin 2 cat. #AF2229 (R&D Systems), rabbit-anti-ATF-2 cat. #35031, rabbit-anti-phospho-ATF-2 cat. #9221 (pT71), rabbit-anti-phospho-VEGFR2-Y1175 (pY1175) cat. #2478 (Cell Signaling Technology, Danvers, MA, USA), goat anti-VE-cadherin, cat. #sc-6458, mouse-anti-PECAM-1 (CD31) cat. #sc-65260, mouse-anti-Tpl2 cat. #37677 (Santa Cruz Biotechnology, Dallas, TX, USA), mouse-anti-PVR (SKII.4) cat. #132202 (BioLegend, San Diego, CA, USA), mouse-anti-α-tubulin, (Sigma-Aldrich, Poole, UK). Endothelial cell growth medium (ECGM) cat. #C-22110 and recombinant human VEGF-A165 cat. #64423 was from PromoCell (Heidelberg, Germany). Tpl2 kinase inhibitor (CAS 871307-18-5) cat. #616373 was purchased from Calbiochem (San Diego, CA USA), ERK1/2 (PD98059) cat. #P-4313, p38 MAPK (SB203580) cat. #S-3400 and JNK (SP600125) cat. #S-7979 inhibitors were purchased from LC Laboratories (Woburn, MA, USA) and Akt (MK2206) cat. #A10003 inhibitor was purchased from AdooQ BioScience (Irvine, CA, USA).

### Cell culture and immunoblotting analysis

Human umbilical vein endothelial cells (HUVECs; PromoCell, Heidelberg, Germany) were grown and characterized as previously described (Fearnley et al., 2014b). Cells were seeded into six-well plates and cultured in ECGM until ∼80% confluent, cells were washed twice with PBS and starved in ECGM for 24 h. If required HUVECs were treated with the desired concentration of a small molecule kinase inhibitor or stimulated with VEGF-A (25 ng/ml). Cells were washed twice with ice-cold PBS, lysed in 2% (w/v) SDS, TBS, 1 mM PMSF and protease inhibitor cocktail cat. #P8340 (Sigma-Aldrich). Protein concentration was determined using the bicinchoninic acid (BCA) assay cat. #23227 (Thermo Fisher Scientific, Loughborough, UK). 20 µg of protein lysate was subjected to SDS-PAGE before transfer onto nitrocellulose membrane and analysis via immunoblotting.

### Quantitative reverse transcription polymerase chain reaction (qRT-PCR)

HUVECs were serum starved for 2 h prior to stimulation with VEGF-A (25 ng/ml) for 1.5 h or 3 h. Total RNA was then extracted using RNeasy Plus Mini Kit cat. #74134 (Qiagen, UK). 1 µg total RNA was reverse transcribed using GoScript Reverse Transcription System cat. #A6001 (Promega, UK). Real time quantitative reverse transcription PCR was performed using Power SYBR Green master mix cat. #4368702 (Applied Biosystems, Warrington, UK) with following primer sets: GAPDH (endogenous control), forward primer 5′–GTC TCC TCT GAC TTC AAC AGC G−3′, reverse primer 5′–ACC CTG TTG CTG TAG CCA A−3′; Tpl2, forward primer 5′–CGC AAG AGG CTG AGT A−3′, reverse primer 5′–TTC CTG TGC ACG AAG AAT CA−3′. qRT-PCR was carried out in multiwell plates run on an ABI 7900HT Fast Real-Time PCR System (Applied Biosystems). Gene expression was analyzed using the delta-delta Ct method standardized against an endogenous control, GAPDH.

### Lipid-based transfection of siRNA duplexes

HUVECs were transfected with siRNA duplexes using Lipofectamine RNAiMAX cat. #13778075 (Thermo Fisher Scientific). Per well of a six-well plate, 15 µl of 2 µM siRNA duplexes (20 nM final concentration) were added to 481 µl of serum/antibiotic-free OptiMEM cat. #31985070 (Thermo Fisher Scientific), briefly inverted and incubated at room temperature (RTP) for 5 min. 4 µl of Lipofectamine was then added and the mixture inverted briefly, before incubation at RTP for 20 min. 2.5×10^5^ cells/ml were seeded in 1 ml volume of OptiMEM, followed by immediate dropwise addition of the siRNA/lipofectamine mixture. Cells were left at RTP for 30 min before being returned to the incubator. After 6 h, media was replaced for ECGM. Cells were allowed to recover for 72 h prior to treatment or processing for analysis.

### Tubulogenesis assay

Primary human foreskin fibroblasts cat. #C-12350 (Promocell) were cultured in 48-well plates in DMEM containing 10% (v/v) FCS, 1% (v/v) non-essential amino acids and 1% (v/v) sodium pyruvate until confluent. 7500 HUVECs were seeded onto the fibroblasts monolayer in a 1 ml 1:1 mixture of DMEM containing 10% (v/v) FCS, 1% (v/v) non-essential amino acids and 1% (v/v) sodium pyruvate and ECGM. Cells were left to acclimatize for 24 h. Media was aspirated and replaced with fresh ECGM ±VEGF-A (25 ng/ml) ±Tpl2 kinase inhibitor (2 µM) as desired; media was replaced every 2–3 days for 7 days. Co-cultures were fixed in 200 µl 10% (v/v) formalin for 20 min and blocked in 5% (w/v) BSA for 30 min at RTP. Co-cultures were then incubated with 1 µg/ml mouse anti-human PECAM-1 (CD31) overnight at 4°C. Cells were washed three times with PBS prior to incubation with donkey anti-mouse Alexa Fluor 594 conjugate cat. #R37115 (Thermo Fisher Scientific) for 3 h at RTP. Wells were washed three times with PBS. Endothelial tubules were visualized via immunofluorescence microscopy using an EVOS-fl inverted digital microscope (Thermo Fisher Scientific). Three random fields were imaged per well. Total tubule length was then quantified from each photographic field using the open source software AngioQuant (www.cs.tut.fi/sgn/csb/angioquant) and values averaged. For a more detailed method please see Fearnley et al. (2014b).

### Leukocyte binding assay

2×10^5^ HL-60 leukocytes/well were labelled with 0.5 µg/ml calcein cat. #C3100MP (Thermo Fisher Scientific) for 30 min at 37°C. Cells were pelleted and washed twice in 5 ml RPMI+10% (v/v) FCS. De-esterification of the calcein reagent was then left to occur for 30 min at 37°C. Cells were then incubated with 100 nM phorbol 12-myristate 13-acetate cat. #P1585 (PMA; Sigma-Aldrich) for 30 min at 37°C. Cells were pelleted and washed twice in 5 ml RPMI. 2×10^5^ HL-60 leukocytes/well were then added onto a confluent HUVEC monolayer, which had been previously stimulated with full growth media (±25 ng/ml VEGF-A for 7 h) and left to adhere for 1 h at 37°C. Non-adhered leukocytes were removed via gentle rinsing with PBS. Bound leukocytes were visualized and photographed via immunofluorescence microscopy using an EVOS-fl inverted digital microscope, before cells were lysed in 200 µl RIPA buffer. 50 µl of cell lysate was then analyzed via fluorescence excitation at 488 nm and emission at 520 nm in a multiwell plate format using a 96-well FLUOstar OPTIMA florescence plate reader (BMG LABTECH, Buckinghamshire, UK). Values were compared to non-stimulated controls.

### MTS cell viability assay

3000 HUVECs were seeded per well of a 96-well plate and cultured in 90 µl ECGM overnight. 10 µl of MTS reagent (CellTiter 96 AQueous Non-Radioactive Cell Proliferation Assay cat. #G5421; Promega, Madison, USA) was added to each well after 20 h. After further incubation for 4 h, we monitored the color change caused by reduction of the yellow tetrazolium compound (MTS) by metabolically active cells to brown formazan. Change in color was monitored at 490 nm using a Tecan Sunrise multi-wavelength 96-well plate reader (Mannedorf, Switzerland).

### *Ex vivo* aortic sprouting assay

All procedures involving animals and their tissues were carried out in accordance to UK Home Office regulations and guidance at RTP unless otherwise stated. Male 8–9-week-old wild-type C57BL/6 mice were euthanized and the thoracic aorta was harvested from aortic arch to diaphragm. Cleaned aortas were sliced into 0.5 mm rings and serum starved overnight at 37°C in 5 ml OptiMEM containing penicillin-streptomycin. Aortic rings were embedded into 55 µl of type 1 rat tail collagen cat. #08-115 (Millipore, Watford, UK) in 96-well plates and stimulated with 150 µl OptiMEM containing 2.5% (v/v) FCS and penicillin-streptomycin ±VEGF-A (25 ng/ml), in the presence or absence of a Tpl2 kinase inhibitor (2 µM). Aortic rings were incubated at 37°C for 5–7 days with a media change on day 3 and 5. Rings were then washed, permeabilized and blocked as previously described (Baker et al., 2012). 2.5 µg BS-I lectin-FITC cat. #L2895 (Sigma-Aldrich) in PBLEC was then added per well and incubated 6 h at RTP. 1 µg/ml DAPI (in PBLEC) was then added per well for 2 h at 37°C. Aortic sprouts were the visualized and imaged using an EVOS-fl inverted digital microscope. Number of initial sprouts (vascular sprouts emanating directly from the aortic ring) were counted and average.

### Assessment of endothelial monolayer permeability using trans-endothelial electrical resistance (TEER)

Human endothelial cells were seeded in 450 µl at 1.5×10^5^ cells/well (sufficient to give a monolayer) into a 0.4 µm pore size Corning Transwell filter cat. #CLS3397 (Sigma-Aldrich) inserted into a 24-well plate containing 500 µl ECGM and left to adhere overnight. At t=0 h the trans-endothelial electrical resistance (TEER) across each monolayer was measured using Millicell-ERS device cat. #MERS00002 (Millipore). Then 50 µl of media MCDB131+0.2% (w/v) BSA ±VEGF-A (25 ng/ml) was added to the upper chamber. After a further 1, 2, 4, 6 or 8 h the TEER across each monolayer was measured again and the relative increase in permeability (corresponding to a decrease in electrical resistance across the endothelial monolayer) was calculated as follows: relative TEER (%)=(TEER of sample of interest–TEER of blank (TEER across insert with no cells)/TEER of control cells at 0 h–blank)×100.

### Statistical analysis of experimental data

This was performed using a one-way analysis of variance (ANOVA) followed by Tukey's post-hoc test or two-way ANOVA followed by Bonferroni multiple comparison test using GraphPad Prism software (La Jolla, USA). Significant differences between control and test groups were evaluated with *P*-values less than 0.05 (*), 0.01 (**), 0.001 (***) and 0.0001 (****) indicated on the graphs. Error bars in graphs and histograms denote ±s.e.m.

## Supplementary Material

Supplementary information
