# Supplementary material for: Tpl2 is required for VEGF-A-stimulated signal transduction and endothelial cell function
Source: Biol Open. 2019 May 9;8(5):bio034215. doi: 10.1242/bio.034215 (PMC6550078; doi:10.1242/bio.034215)
Supplement: Supplementary information [file biolopen-8-034215-s1.pdf]

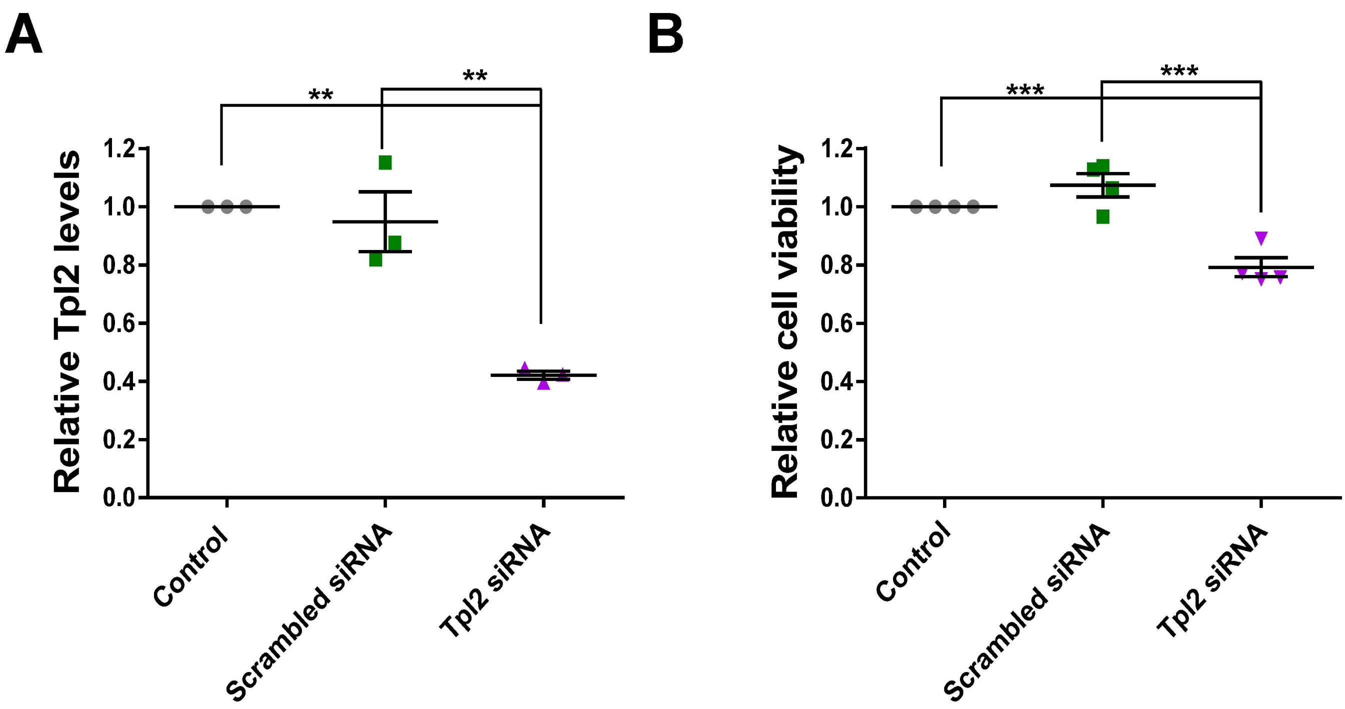

Fig. S1: Depletion of endothelial Tpl2 affects cell viability. (A). Quantification of immunoblot data showing relative Tpl2 levels. Error bars indicate ±SEM (n=3). Statistical test used was one-way ANOVA; p<0.01 (**). (B) Assessment of endothelial cell viability for control, scrambled siRNA-treated or Tpl2-depleted endothelial cells. Error bars indicate ±SEM (n=4). Statistical test used was one-way ANOVA; p<0.001 (***).

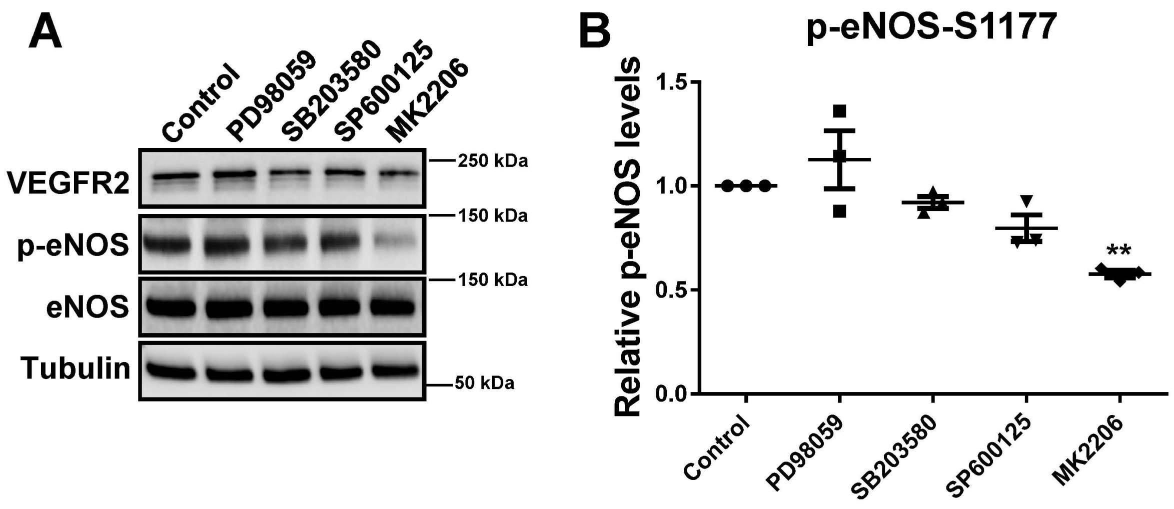

Fig. S2: Inhibition of basal Akt kinase activity reduces eNOS phosphorylation. (A) Immunoblot analysis of endothelial cells treated with small molecule kinase inhibitors (2 µM; 30 min) specific for ERK1/2 (PD98059), p38 MAPK (SB203580), JNK (SP600125) and Akt (MK2206). (B) Quantification of relative levels of eNOS-pS1177 upon pharmacological inhibition of different protein kinases from immunoblot data. Error bars indicate ±SEM (n=3). Statistical test used is one-way ANOVA; $p<0.001$ (***).

Biology Open • Supplementary information
